# Dynamic Culture of Mesenchymal Stromal/Stem Cell Spheroids and Secretion of Paracrine Factors

**DOI:** 10.3389/fbioe.2022.916229

**Published:** 2022-08-15

**Authors:** Paloma Fuentes, María José Torres, Rodrigo Arancibia, Francisco Aulestia, Mauricio Vergara, Flavio Carrión, Nelson Osses, Claudia Altamirano

**Affiliations:** ^1^ Escuela de Ingeniería Bioquímica, Facultad de Ingeniería, Pontificia Universidad Católica de Valparaíso, Valparaíso, Chile; ^2^ Cellus Medicina Regenerativa S.A., Santiago, Chile; ^3^ Cellus Biomédica, Parque Tecnológico de León, León, Spain; ^4^ Departamento de Investigación, Postgrado y Educación Continua (DIPEC), Facultad de Ciencias de la Salud, Universidad del Alba, Santiago, Chile; ^5^ Instituto de Química, Pontificia Universidad Católica de Valparaíso, Valparaíso, Chile; ^6^ CREAS, Centro Regional de Estudios en Alimentos Saludables, Valparaíso, Chile

**Keywords:** MSC, conditioned medium, spheroids, dynamic culture, paracrine factors, secretome

## Abstract

In recent years, conditioned medium (CM) obtained from the culture of mesenchymal stromal/stem cells (MSCs) has been shown to effectively promote tissue repair and modulate the immune response *in vitro* and in different animal models, with potential for application in regenerative medicine. Using CM offers multiple advantages over the implantation of MSCs themselves: 1) simpler storage, transport, and preservation requirements, 2) avoidance of the inherent risks of cell transplantation, and 3) potential application as a ready-to-go biologic product. For these reasons, a large amount of MSCs research has focused on the characterization of the obtained CM, including soluble trophic factors and vesicles, preconditioning strategies for enhancing paracrine secretion, such as hypoxia, a three-dimensional (3D) environment, and biochemical stimuli, and potential clinical applications. *In vitro* preconditioning strategies can increase the viability, proliferation, and paracrine properties of MSCs and therefore improve the therapeutic potential of the cells and their derived products. Specifically, dynamic cultivation conditions, such as fluid flow and 3D aggregate culture, substantially impact cellular behaviour. Increased levels of growth factors and cytokines were observed in 3D cultures of MSC grown on orbital or rotatory shaking platforms, in stirred systems, such as spinner flasks or stirred tank reactors, and in microgravity bioreactors. However, only a few studies have established dynamic culture conditions and protocols for 3D aggregate cultivation of MSCs as a scalable and reproducible strategy for CM production. This review summarizes significant advances into the upstream processing, mainly the dynamic generation and cultivation of MSC aggregates, for de CM manufacture and focuses on the standardization of the soluble factor production.

## GRAPHICAL ABSTRACT

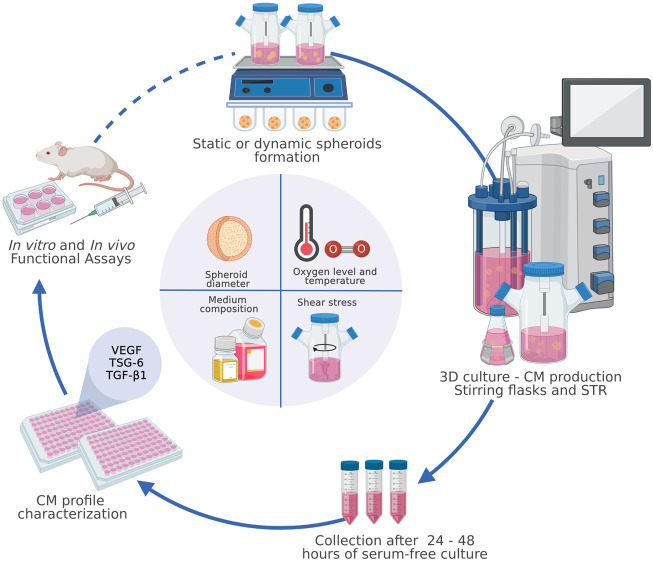

## 1 Introduction

Mesenchymal stromal/stem cells (MSCs) were described for the first time in 1970 by Friedenstein as a population of bone marrow stromal cells capable of mesodermal differentiation and trophic support of hematopoiesis (Friedenstein, et al., 1970). In 2006, the International Society for Cellular Therapy (ISCT) established “minimal” criteria for identifying MSCs. These criteria include trilineage differentiation potential (the ability to differentiate into adipocytes, chondrocytes, and osteocytes); cell-surface expression of mesodermal markers such as CD90, CD105, and CD73; and a lack of expression of hematopoietic markers such as CD45, CD34, CD14, CD19 and HLA-DR (Dominici et al., 2006). MSCs reside in almost all postnatal organs and tissues (da Silva Meirelles et al., 2006). They can be isolated from the bone marrow (Gnecchi and Melo 2009), adipose tissue (Zuk et al., 2001), umbilical cord (Gimble et al., 2007), placenta (González PL et al., 2015), menstrual blood (Luz-Crawford et al., 2016), and dental pulp (Perry et al., 2008).

Until 2008, clinically administered MSCs were almost entirely bone marrow derived. Today, the diversification of sources of tissue and MSC products, with more than 1,000 clinical studies registered for “mesenchymal stem cells” and at least 200 registered for “mesenchymal stromal cells” (www.clinicaltrials.com), has promoted the establishment of guidelines, with definitions of minimal criteria, by several regulatory authorities. Some of these guidelines have proposed the incorporation of TF/CD142 expression and hemocompatibility assessment as new markers/criteria for MSCs intended for intravascular use, with the aim of ensuring the quality, potency and safety of these therapies (Moll et al., 2019; Moll et al., 2022).

Due to their characteristics, MSCs are considered a powerful and versatile tool in cell therapy and tissue engineering applications. The first studies in this area emphasized their regenerative properties through the engraftment, replacement, and renewal of damaged tissues (Muñoz-Elias et al., 2004; Amado et al., 2005; Sasaki et al., 2007; Wu et al., 2007). Moreover, MSCs have emerged as critical modulators of innate and adaptive immune responses (Ma et al., 2014; Xie et al., 2014; Luk F et al., 2015; Gao et al., 2016). They have been used to treat autoimmune and inflammation-associated diseases, including graft-versus-host disease (Le Blanc et al., 2008), rheumatoid arthritis (Tanaka 2015), multiple sclerosis (Cohen, Imrey et al., 2018), osteoarthritis (OA) (Pers et al., 2016), and systemic lupus erythematosus (Wang et al., 2014), among other serious complications, such as hemorrhagic cystitis (Tong et al., 2020) and acute respiratory distress syndrome (Hamdan et al., 2021; Ringdén et al., 2022). The cellular and molecular mechanisms underlying the therapeutic properties of MSCs are still not completely understood. The demonstrated *in vitro* multipotential differentiation of MSCs contrasts with the limited long-term engraftment and survival of transplanted cells observed *in vivo* (Von Bahr et al., 2012), which can be partly attributed to the induction of an immediate blood-mediated inflammatory reaction upon infusion of MSC products that are not fully compatible with human blood (Moll et al., 2012) and by the extensive use of frozen cells (Moll et al., 2014). A typical therapeutic dose of 100 million cells is reduced to less than 1% detectable cells in the target tissue (Pittenger et al., 2019). However, this dose induces a beneficial effect beyond the small number of cells actively replaced by direct differentiation. This strongly suggests that tissue repair is mediated by soluble factors and microvesicles that MSCs secrete into the extracellular environment (Caplan and Dennis 2006; Gnecchi et al., 2008; Doorn et al., 2012; Liang et al., 2014).

Conventionally, MSCs have been expanded and cultured in two-dimensional (2D) cultures as monolayers attached to planar polystyrene flasks. This strategy has been widely used because of its easy implementation and low associated costs. Thus, the manufacturing process required to produce trillions of cells has been scaled up in multilayered cell culture flasks or cell factories (Rowley, 2012). However, 2D culture has inherent technical limitations in terms of scale, monitoring, control, and restrictions on cell–cell and cell-matrix interactions. Moreover, this labour-intensive approach is not a closed and automated process, and it may not be satisfactory for cost-effective therapies (Panchalingam et al., 2015). The rigid and artificial conditions used for these systems alter the cellular communication that occurs in the cell’s physiological niche (Duval et al., 2017), thus affecting not only the arrangement and morphology of the cells but also their capacities for proliferation, differentiation, and secretion of paracrine factors (Bara et al., 2014). In this context, the expansion of MSCs in a controlled and reproducible way in a suspension bioreactor system represents a scalable alternative to static 2D culture.

In recent years, the culture of scaffold-free MSCs in bioreactors has emerged as a promising bioprocess for industrial purposes. This strategy harnesses the spontaneous ability of single dispersed cells to self-assemble into spherical multicellular aggregates. The culture of MSCs in such a three-dimensional spatial configuration is known as three-dimensional (3D) culture, and the generated multicellular aggregates are known as spheroids (Cesarz and Tamama 2016). MSC aggregates preserve and enhance the secretory capacity of the cells, enabling the production of cell-free products with therapeutic potential, like conditioned medium (CM) (Bartosh, Ylöstalo et al., 2013; Zimmermann and Mcdevitt, 2014; Kwon, Bhang et al., 2015; Santos, Camoes et al., 2015; Miranda, Camoes et al., 2019). CM is defined as all the secreted factors and microvesicles found in the medium in which the MSCs were cultured (Pawitan 2014). In the literature, few works characterize the CM and do so in an unexhaustive manner. In addition, the different components identified are usually related to the particular research objective, and there are no studies that carry out a large-scale analysis of the components of the CM. However, *in vitro* studies and animal models of different pathologies have demonstrated the properties and regenerative potential of this product. This review aims to present the advances and challenges in spheroids MSC culture and CM production from an integrated bioprocess perspective.

## 2 Definitions and Advantages of Mesenchymal Stromal/Stem cells Culture as Multicellular Aggregates

Traditionally, *in vitro* expansion of MSCs has been performed in culture flasks in which the cells grow attached to a plastic surface *via* adsorption of molecules present in the extracellular matrix (Cesarz and Tamama 2016). Under this strategy, cells grow, forming a flat, 2D monolayer, which is commonly referred to as a 2D culture. According to this classification, a 3D culture of multicellular aggregates is one in which the cells adopt a three-dimensional spatial configuration. This conformation better resembles the conditions observed in the physiological environment, where intercellular junctions, the formation of cell-matrix complexes, and the existence of molecular gradients towards the centre of the aggregate predominate (Achilli, Meyer et al., 2012). All these factors favour the establishment of interactions involved in signalling processes, communication, and cellular secretion of bioactive factors (Petrenko, Syková et al., 2017; Kouroupis and Correa 2021).

Different strategies have been developed for the generation of multicellular aggregates.

These strategies are classified into two types: 2.1) the formation of supported multicellular aggregates, which involves immobilization techniques such as microcarriers, hydrogels, and encapsulation systems for the formation and stabilization of multiaggregates; and 2.2) the formation of support-free cellular multiaggregates, which is based on the ability of MSCs to spontaneously group and generate multicellular aggregates under specific culture conditions without the need for external support. Both techniques have been fundamental in the transition from the 2D culture paradigm to 3D culture of MSCs, so the following section will review their advantages and disadvantages.

### 2.1 Scaffold-Based Mesenchymal Stromal/Stem Cells Aggregates

Regarding immobilization techniques, both the nature of the material chosen (synthetic or natural) and the intrinsic properties of the material, including its porosity, rigidity, and polarity, impact cell behaviour. Thus, proliferation, migration, phenotype, and differentiation potential are affected (Zhao, Li et al., 2014; Mizukami, Fernandes-Platzgummer et al., 2016; Mizukami and Swiech 2018). Many types of microcarriers are commercially available, but the most typical microcarriers consist of polystyrene, dextran, or gelatine (Kong, Chen et al., 1999). The selection of an appropriate microcarrier is critical for designing a production process since cell harvesting by detachment must not alter the viability and qualitative characteristics of the MSCs (Nienow et al., 2014; Mizukami, Fernandes-Platzgummer et al., 2016). However, the use of scaffolds has some disadvantages beyond the technical complexities of detaching and harvesting the cells. For example, one relevant aspect of the production of CM and derived products is that by using an external matrix in the formation of the aggregates, the information flow at the molecular level is not as dynamic and biologically complex as the signal exchange that occurs when cells are in direct contact (Achilli, Meyer et al., 2012). This limitation occurs because the use of scaffolds means that both cells and extracellular matrix (ECM) molecules continue to adhere to an external surface (the foundation of 2D culture), even though a 3D configuration exists, making it challenging to recapitulate the intimate cell–cell and cell-matrix architecture found in physiological tissues (Antoni, Burckel et al., 2015).

It should also be noted that scaffold stiffness and topography influence the differentiation potential of human MSCs (Engler, Sen et al., 2006; Hupfeld, Gorr et al., 2014; Zhao, Li et al., 2014); thus, a microcarrier’s mechanical properties induce differentiation towards specific lineages. Cell adhesion to a surface carrier requires glycoproteins and serum components that also play a cell-protective role, so the presence of the microcarrier at the beginning of the culture process is often needed. However, the massive volumes of fetal bovine serum (FBS) needed for the large-scale production of cellular products make this manufacturing process expensive. To comply with regulations, such as European Medicines Agency (EMA) guidelines (see the EMA ‘Note for Guidance on the Use of Bovine Serum in the Manufacture of Human Medicinal Products’ at http://www.ema.europa.eu), it is necessary to use FBS certified in terms of its origin, preparation, safety, pathogen inactivation ability, and particle-free nature. All these analyses contribute to the high price of FBS.

### 2.2 Scaffold-Free Mesenchymal Stromal/Stem Cells Aggregates

While the term cellular multiaggregate refers to a particle made up of a group of compacted cells, the term spheroid refers specifically to a group of cells forming a spherical particle primarily associated with support-free multicellular aggregates (Achilli, Meyer et al., 2012; Costa, McDevitt et al., 2017; Petrenko, Syková et al., 2017). In this review, the term spheroids will refer exclusively to scaffold-free multicellular aggregates.

Studies focused on the formation and culture of cells as spheroids began over 70 years ago with embryonic cell research (Mueller-Klieser 1987). Later, spheroids were established as a validated method for cancer studies (Hamilton 1998). The implementation and optimization of spheroids are currently of great interest, with a particular emphasis on tissue engineering and cell therapy, and more recently, interest in developing new therapeutic strategies based on cell-free products has grown (Hamilton 1998; Potapova, Gaudette et al., 2007). Different research groups have demonstrated the multiple benefits of spheroids, ranging from improvements at the genetic, metabolic, and functional levels (Sart, Tsai et al., 2014; Tsai, Yang et al., 2019) to logistical aspects related to storage and transport (Jiang, Yan et al., 2017).

Cultured as spheroids exhibit stronger stemness properties than MSCs grown in 2D culture. This increase is evidenced by the overexpression of pluripotency markers such as Oct4, Nanog, Sox2, and Rex1 (Cheng et al., 2013), increased differentiation potential, and higher colony formation efficiency, without the need to add proteins to the culture medium as stimuli (Zhang et al., 2015). Similarly, it has been shown that under this configuration, MSCs show more marked cellular plasticity than that observed in 2D cultures in response to epigenetic changes that favour cell differentiation towards lineages other than mesodermal, such as neuronal cells (Guo et al., 2014). In addition to the enhanced differentiation and transdifferentiation capacity of these cells (García-Sánchez et al., 2019), when evaluating different adipogenic induction protocols, spheroids have been shown to require a much shorter stimulus time than MSCs cultured in 2D culture to trigger and maintain the signalling cascade involved in the differentiation process (Hoefner et al., 2020). Spheroids exhibit better functional abilities in a shorter time, which is attractive for industrial applications (Ezquerra et al., 2021).

Additionally, spheroid culture is positioned as an attractive strategy for developing therapeutics and derived products. The configuration of spheroids provides cell–cell and crosstalk contact without external intermediaries, simulating the interactions orchestrated by MSCs in their *in vivo* niche (Chimenti, Massai et al., 2017). Because they do not require microcarriers, encapsulation technologies, or adhesion molecules for their formation or maintenance, scaffold-free spheroids are associated with lower production costs (Jenkins and Farid 2015) and fewer culture components, facilitating subsequent development and validation under good manufacturing practice (GMP) processes (Kropp, Kempf et al., 2016).

## 3 Methods for Spheroid Formation

**FIGURE 1:**
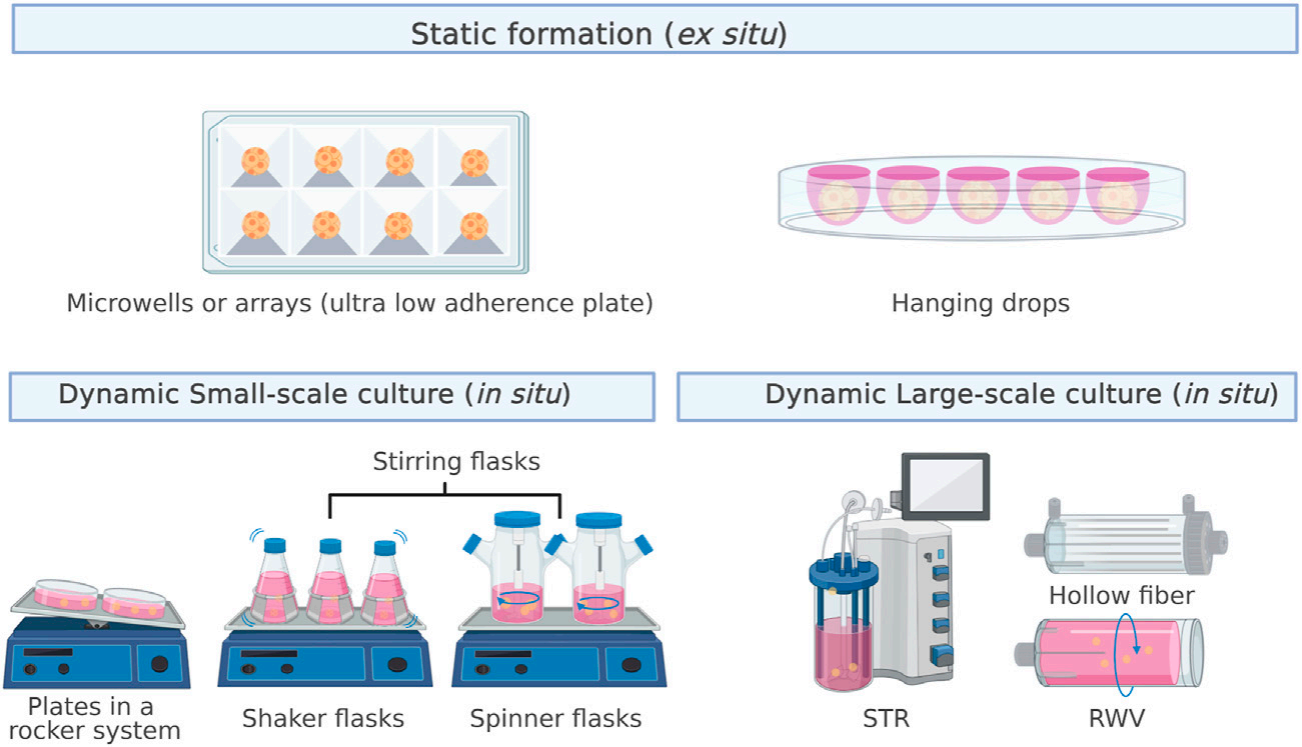
Strategies for static (*ex situ* culture) and dynamic *(in situ* culture) spheroid formation. The most used approaches for static formation, also called MSCs self-assembly, are microwells and the hanging drop method. In contrast, dynamic formation, also called collision-based assembly, can occur in small- or large-scale cultures.

The formation of spheroids from a single-cell suspension occurs mainly through two processes: cluster-based self-assembly and collision-based assembly (Achilli, Meyer et al., 2012). In the former, segregated cells settle into compartments that promote cell–cell interactions, leading to the formation of multicellular spheroids, similar to the natural development process that drives organogenesis. In cell culture, this process occurs in a nonstirring environment through the joint action of surface tension and gravitational force, which is why the cultures are referred to as static cultures (Egger, Tripisciano et al., 2018). Hanging drops, low-adhesion surfaces, or external forces, such as centrifugal force and magnetic fields, are used to promote aggregation. In collision-based self-assembly, single cells collide through continuous stirring to promote aggregation, which generates a dynamic suspension culture (Egger, Tripisciano et al., 2018). Figure 1 shows the leading strategies for static (*ex situ* culture) and dynamic *(in situ* culture) spheroid formation.

### 3.1 Cluster-Based Self-Assembly/Static Formation

For static formation, MSC aggregation is typically induced by hanging drops or culture on microplates. MSC culture in microplates originates small spheroids of 300–500 cells, with diameters between 125 and 200 μm, in 12–48 h. In comparison, in the hanging drop approach, larger spheroids, with a diameter of 200–400 μm, are formed within 24–48 h by plating drops with 30,000 or 50,000 cells (Hildebrandt, Büth et al., 2011; Bhang, Lee et al., 2012; Cho, Song et al., 2012; Bhang, Lee et al., 2014). Among the advantages of static aggregation are its simple protocol, lack of requirement for specialized equipment, and ability to control the number of cells per spheroid, which leads to a uniform size (Frith, Thomson et al., 2010; Bhang, Cho et al., 2011; Hildebrandt, Büth et al., 2011; Bhang, Lee et al., 2014; Han, Asano et al., 2019; Ryu, Lee et al., 2019). However, this strategy is tedious in terms of cell handling and medium exchange, time-consuming, dependent on long-term culture, low throughput, and nonscalable. To overcome some of these drawbacks, automated microfluidic platforms have emerged, allowing the generation of many spheroids per array and even programmed renewal of small volumes of culture medium (Huang, Tzeng et al., 2020). Although these alternatives are promising as *in vitro* models for the study of differentiation processes, for drug testing and, to a lesser extent, for the generation of therapeutic cell products, they remain limited for the development of strategies for the large-scale production of conditioned medium and cell-free products.

### 3.2 Cluster-Based Self-Assembly/Dynamic Formation

Different systems have been used for spheroid formation under dynamic conditions, including the use of shaking plates and stirring flasks (such as shake and spinner flasks) (Bhang, Cho et al., 2011; Alimperti, Lei et al., 2014; Kwon, Bhang et al., 2015; Li, Guo et al., 2015; Miranda, Camoes et al., 2019) and the use of bioreactor systems (Zhang, Liu et al., 2015; Egger, Schwedhelm et al., 2017). The formation of small spheroids (diameter of less than 50 µm) in less than 24 h was observed from umbilical cord MSCs seeded on glass plates with continuous shaking at 10 rpm. However, studies performed with stirring flasks reported differences in the number of days required for spheroid formation. Alimperti et al., 2014 reported spheroid formation after overnight culture (12–24 h), without agitation, from a suspension at a density of 1 × 10^5^ cells/ml using umbilical cord human MSCs at passage 4 in serum-free medium (Alimperti, Lei et al., 2014). In contrast, Niibe et al., 2020 observed spheroid formation after 14 days, for both cultures starting at 5 × 10^4^ cells/ml and those starting at 5 × 10^5^ cells/ml, using the same cell type at passages four to seven but with stirring at 85–95 rpm from time 0 in αMEM containing 10% FBS (Niibe, Ohori-Morita et al., 2020). Other studies have shown that the formation of spheroids in stirring flasks requires 24–72 h, depending on the desired compaction, with agitation speeds fluctuating between 70 and 110 rpm to ensure that the spheroid diameter is less than 350 µm (Bhang, Cho et al., 2011; Kwon, Bhang et al., 2015; Santos, Camoes et al., 2015; Miranda, Camoes et al., 2019). However, a recent study showed that it is only possible to measure spheroid diameter consistently after 6 days of culture in a stirring flask since highly heterogeneous aggregates are generated in the first days of culture (Allen, Matyas et al., 2019). This heterogeneity also occurs because there is no consensus protocol or clearly defined parameters for determining spheroid quality in morphological and functional terms. Therefore, differences in compactness, diameter, circularity, number of cells per aggregate, and percentage efficiency (number of spheroids formed from the number of seeded cells) have been reported for spheroids that are considered valid.

Only two studies on spheroid formation in bioreactors have been published to date. The first has been performed in a rotating wall vessel bioreactor, also called a microgravitational bioreactor. The cultures in this study started at a density of 1 × 10^6^ cells/ml at 25 rpm, achieving aggregation of cells in 24 h, with an average spheroid diameter close to 80 µm (Zhang, Liu et al., 2015). However, the number of spheroids formed was not quantified. The second, the only study performed in a stirred tank reactor reported the formation of visible spheroids after 3 days of stirred culture at 600 rpm from a cell suspension at 1 × 10^5^ cells/ml (Egger, Schwedhelm et al., 2017). However, in this case, no parameters related to the morphology and structure of the obtained aggregates were reported.

Overall, the results obtained to date show that the spheroids formed under dynamic conditions are characterized by a heterogeneous size and formation time, unlike those produced in microplates. However, recent studies have shown that it is feasible to control spheroid size through agitation speed and shear stress, as reviewed in the following section. Additionally, these systems have several advantages that become more relevant when the aim is to produce CM or cell-free products; this is especially true for systems that allow online monitoring and control of the operational conditions, such as bioreactors.

## 4 Dynamic Culture of Spheroids and Conditioned Medium Production

Dynamic culture represents an excellent alternative for cell expansion and the development of strategies for the generation of cell-free products and therapies, as it provides a more homogeneous environment than culture under static conditions. A dynamic culture ensures better diffusion of nutrients, signalling molecules, waste metabolites, and oxygen. Dynamic culture allows spheroid cultures to be sustained for extended periods (up to 2 months) (Niibe, Ohori-Morita et al., 2020), maintaining morphological parameters and the reattachment capacity of spheroid-derived MSCs. As a result, the cells preserve their transdifferentiation potential and support cell growth at a density approximately four times higher than the density of a 2D culture (Bhang, Lee et al., 2014; Niibe, Ohori-Morita et al., 2020). This culture strategy is a promising option for CM production. It increases the concentration of growth factors in the supernatant and induces the secretion of specific angiogenic, pro-regenerative, anti-inflammatory, or anti-apoptotic mediators. In addition, the secretory profile of the CM and its different therapeutic targets can be driven by and depends on manipulating specific operational parameters (Zimmermann and Mcdevitt, 2014; Miranda, Camoes et al., 2019). Recently, various strategies have been designed and evaluated for the dynamic culture of MSC spheroids; these strategies consist mainly of processes similar to those mentioned for the dynamic formation of spheroids.

These strategies can be divided into two groups: small-scale culture (Section 4.1) in plates and stirring flasks and large-scale culture (Section 4.2) in bioreactor systems. The first group involves culture plates and flasks that require the use of incubators for temperature, humidity, oxygen, and CO_2_ control, as well as agitation platforms, with either orbital agitation (for shaking plates and flasks) or magnetic agitation (in the case of spinner systems; magnetic impeller stirring). Because of these additional requirements and because these devices rarely have systems for online monitoring and control of operational variables, plate and shaking flask cultures are low-scale systems. In contrast, bioreactors with mechanical impeller stirring and the ability to monitor and control integrated variables are large-scale systems since they also allow direct scaling up to different working volumes.

### 4.1 Small-Scale Culture: Plates and Stirring Flasks

The implementation of small-scale dynamic culture has been successful at the research level. It has been shown to maintain the multipotentiality of MSCs, enhance differentiation potential, and increase the secretion of paracrine factors. This culture strategy requires, in most cases, two steps: the formation of spheroids by a static method and subsequent inoculation and culture of the spheroids in a dynamic system. Aggregate formation does not take place in the same culture device or under the same conditions (Hildebrandt, Büth et al., 2011; Cha, Shin et al., 2018), which is significantly different from spheroid formation under fully dynamic conditions. For example, spheroids of murine MSCs formed on Aggrewell plates and subsequently cultured on plates with orbital shaking at 30 rpm have been shown to maintain an undifferentiated state for up to 16 days (Baraniak and McDevitt 2012). Using this approach, spheroids of 500 cells/aggregate grown in suspension at 65 rpm on low-adherence plates show 89 ± 5% viability after 4 days of culture, similar to 2D culture, with increased expression of proangiogenic cytokines and increased resistance to oxidative stress compared to those of 2D MSCs and encapsulated MSC aggregates (Costa, McDevitt et al., 2017). In terms of functionality, spheroids of MSCs in serum-free medium cultured with continuous agitation in a rocker system at 10 rpm have been shown to promote tissue repair in an *in vivo* model of severe liver damage (Li, Guo et al., 2015).

Recent publications have shown that stirring flasks can be inoculated with both preformed spheroids (*ex situ* formation) and single cells (*in situ* formation), comparable levels of proliferation (Allen, Matyas et al., 2019). Thus, stirring flasks are an interesting alternative for spheroid culture, as they increase the working volume. Additionally, stirring flasks allow greater control of mass transfer and energy because of the convective forces generated in the vessel through orbital or magnetic stirring. It has been reported that the culture conditions generated in stirring flasks do not alter cell viability or proliferation, but they do increase trilineage differentiation (Alimperti, Lei et al., 2014; de Bournonville, Lambrechts et al., 2019). Moreover, MSC spheroids cultured in stirring flasks exhibit lower expression of some surface markers, such as CD105 and CD90, after 7 days of culture, similar to 2D culture (Bartosh, Ylöstalo et al., 2010). Nevertheless, this expression is recovered when the MSCs are plated on a surface (Santos, Camoes et al., 2015).

MSCs cultured in stirring flasks show decreased expression of pro-apoptotic proteins and better preservation of the extracellular matrix composition, maintaining the improved migration and cell adhesion properties of spheroids compared to MSCs cultured in 2D (Bhang et al., 2011). In addition, spheroids obtained by the hanging drop method and subsequently cultured with agitation at 70 rpm showed increased viability compared to that of MSCs grown in 2D culture, with enhanced survival and secretion of paracrine factors after transplantation and in an *in vivo* model of ischaemic limbs (Bhang et al., 2012). These improvements have also been observed in serum-free 3D cultures. Higher cell density has been achieved in 3D cultures than in 2D cultures, with 7 × 105 ± 0.8 cells/ml in a stirring flask with 70 ml of medium versus 3.1 × 105 ± 0.5 cells/ml in a 150 cm^2^ flask with 24 ml of medium (Bhang et al., 2014). However, comparing results between 3D and 2D cultures is not straightforward given the intrinsic differences between the two systems, and it seems necessary to move towards the use of standardized controls for this type of assay.

CM obtained from spheroids cultured in stirring flask exhibits high levels of VEGF, FGF2 and immunosuppressive factors, such as HGF and TGFβ1, which were capable of inducing tissue repair in an animal model of cutaneous wounds (Santos, Camoes et al., 2015). Along the same lines, an increase in the secretion of IL-24, a cytokine related to decreased cancer cell viability, has been observed (Frith, Thomson et al., 2010). Moreover, CM derived from 3D dynamic cultures (in stirring flasks) promoted a 1.5-fold increase in chondrocyte migration capacity 24 h post-scratch in an *in vitro* healing assay, when compared to CM obtained from 2D cultures (Miranda, Camoes et al., 2019). A comparative analysis at the proteomic level, showed high productivities of factors that exert mitogenic, protective and motogenic effects on chondrocytes (PDGFB, IL10, and FGF2) in the CM obtained from 3D cultures. These results are promising for the development of strategies for the generation of cell-free products. However, the use of both culture plates on rotating platforms and stirring flasks is incompatible with a larger production scale. Both approaches use agitation systems that differ from the hydrodynamic profile obtained at higher working volumes, thus hindering the scaling and mixing process (Rodrigues et al., 2018a)

### 4.2 Large-Scale Culture: Bioreactors

The widespread use of bioreactors in the biopharmaceutical industry to produce recombinant proteins in mammalian cell lines has led to the development of essential expertise in bioprocesses. To date, this expertise has been applied for manufacturing stem cell-based products (Rodrigues, Silva et al., 2018). However, compared to conventional mammalian cell lines, the culture of MSCs represents a more significant challenge, as their phenotypic identity and potential must be maintained throughout the culture time. There is accumulating evidence that bioreactors are an efficient system for culturing MSC spheroids (Tostões, Leite et al., 2012). This suitability can be attributed to the fact that bioreactors are equipped with sensors that allow online monitoring and control of crucial culture parameters, including physicochemical variables, such as pH, oxygen tension (OT), and temperature, and biochemical variables, such as the concentrations of nutrients, metabolic byproducts and growth factors (Rodrigues, Fernandes et al., 2011). The increased control of operational variables positions bioreactors as the ideal platform for producing and culturing 3D aggregates (Yeatts, Choquette et al., 2013; Rodrigues et al., 2018a).

### 4.3 Stirred Tank Bioreactors

The STR is the most widely used type of reactor at the industrial level. It comprises a glass or stainless-steel vessel equipped with mechanical impellers, which act as an agitation system generating a dynamic, homogeneous environment that allows the maintenance of cells in suspension. It is easy to scale up and can be used under different culture modalities, such as batch, fed-batch, chemostat, and perfusion culture, making it an attractive system for expanding different cell types in suspension (Nienow 2006). The culture of MSCs in these systems has commonly involved scaffolds (Dos Santos, Campbell et al., 2014; Cunha, Aguiar et al., 2017; Lawson, Kehoe et al., 2017; Mizukami and Swiech 2018). Despite several studies in which the culture of other types of stem cell spheroids has been successfully implemented (Kropp, Kempf et al., 2016; Abecasis, Aguiar et al., 2017), the culture of MSC aggregates in STRs has rarely been investigated.

In fact, in our literature review, there was only one published study performing spheroid culture of MSCs in STRs (Egger, Schwedhelm et al., 2017). This study showed that the conditions generated in an STR at 600 rpm maintain cell viability. In addition, cells from spheroids that were disaggregated with Accumax solution maintained their marker expression and differentiation potential, increasing adipose and chondrogenic differentiation. The results obtained in this work pave the way for further development and optimization of strategies to expand MSCs and collect the produced compounds.

### 4.4 Rotating Wall Vessel Bioreactors

Other bioreactors have also been studied to achieve culture conditions that may favour one phenotype over another in MSCs (dos Santos, Andrade et al., 2013). Such is the case for the use of RWV bioreactors. RWV bioreactors comprise a culture chamber containing an internal cylinder covered by a membrane through which gas transfer occurs. This rotation system generates a microgravitational environment in which the cells remain in “free fall” during the entire culture (Sheyn, Pelled et al., 2010). RWV bioreactors can provide a homogeneous microenvironment at low agitation speeds (15 rpm), allowing the formation and maintenance of aggregates for 7 days, with a diameter that ensures oxygen diffusion (18.1–43.5 µm) and viability comparable to that of cells cultured in 2D culture (Frith, Thomson et al., 2010). The properties of MSCs derived from spheroids cultured in a microgravitational bioreactor at 25 rpm have been studied. The spheroids were disaggregated after 5 days of culture, and the obtained cells were reseeded in 2D culture flasks. After 5 days, these MSCs show increased therapeutic potential in a model of acute liver failure compared to those cultured in monolayers (Zhang, Liu et al., 2015). Although RWV bioreactors provide a more favourable environment for aggregates, their limited volumetric capacity and technical complexity hinder the scalability of the system (Rodrigues, Fernandes et al., 2011; Kropp, Kempf et al., 2016).

Other culture platforms used for large-scale expansion of MSCs combine the use of scaffolds and suspension cultures or bioreactors that provide a surface area for MSCs growth, such as the use of packed bed and hollow fiber bioreactors. In these systems, the cells are “immobilized” on a surface; therefore, they are not considered suspension cultures (Rodrigues et al., 2018a; Rodrigues et al., 2018b; Burns, Doris et al., 2021), which is why they will not be addressed in this review. However, recent advances in noninvasive monitoring of variables in hollow fiber cultures and promising results in the culture of MSCs and other stem cells for extended periods (Mizukami, de Abreu Neto et al., 2018; Greuel et al., 2019) position these bioreactors as an attractive alternative at a commercial level, so further research in this type of system is warranted.

The culture of MSC aggregates in bioreactors provides new opportunities to develop scalable, consistent, and more robust production strategies and reveals a new spectrum of challenges. The need to provide the cells with an adequate oxygen supply must be balanced against the detrimental effects of hydrodynamic shear stress on the particles due to mechanical agitation and aeration (King and Miller 2007). In turn, the impact of this balance on the viability, proliferation, differentiation potential, and secretory profile of MSCs must be evaluated. To successfully implement the dynamic culture of 3D MSCs in bioreactors, it is essential to combine the different operational variables to generate the best likely outcome. For example, it is necessary to determine specific culture conditions (growth medium, supplements, oxygen tension, and temperature) to increase cell viability and proliferation while avoiding differentiation towards particular lineages. Another example is the need to promote the production of a conditioned medium with a secretion profile that yields a specific therapeutic outcome over an increase in proliferation and biomass production. These examples show that the quality of the MSCs and products obtained should always prevail over the productivity that can be achieved. In this scenario, the evaluation of different operating conditions and their effects on the morphology, viability, multipotentiality, differentiation potential, and functionality of the 3D aggregates is crucial to the standardization of both the procedures used and the characteristics of the derived products. Table 1 and 2 shows a comparison of different studies reporting static and dynamic methods of spheroid formation and their results for the dynamic cultivation of MSC spheroids and their impact in CM profile.

**TABLE 1:** Comparison of different studies reporting static and dynamic methods of spheroid formation and their results for the dynamic cultivation of MSC spheroids.

Ref.	MSCs source	Ps	Formation method	Dynamic culture	T°	rpm	O_2_	Medium	Time	Initial cell density	Priming	Cells per spheroid	Spheroid parameters
Frith et al. (2010)	human BM-MSCs	nr.	Static (*ex situ*) Microwells in non-adherent plates	Small- and large-scale SF and RWV	37°C	30 (SF) and 15 (RWV)	nr. 21% ia.	αMEM + 15% FBS + pen/strep	7 days	1 × 10^6^ cells/ml (formation) 2 × 10^4^ cells/ml (culture)	nr.	Random	Diameter 98.7 (SF) and 31.7 µm (RWV)
Bhang et al. (2011)	human AD-MSCs	5	Dynamic (*in situ*) Single-cell inoculation in SF	Small-scale: SF	37°C	70	1 and 5% (culture)	αMEM + 10% FBS + pen/strep	3 days	6 × 10^5^ cells/ml	nr.	Random	Diameter 100–350 µm
Hildebrandt et al. (2011)	human BM-MSCs	3 to 10	Dynamic (*in situ*) Non-adherent bacterial plate on a rotating platform	Small-scale: Bacterial culture dishes on a rotating platform	37°C	75 for 24 h then raised to 85 to 95	nr. 21% ia.	αMEM + 15% FBS + pen/strep	21 days	5 × 10^4^ cells/ml	nr.	Random	Multiaggregation (diameter not reported)
Hildebrandt et al. (2011)	human BM-MSCs	3 to 10	Static (*ex situ*) HP	Small-scale: Bacterial culture dishes on a rotating platform	37°C	75 for 24 h then raised to 85 to 95	nr. 21% ia.	αMEM + 15% FBS + pen/strep	21 days	5 × 10^3^ cells per drop (formation)	nr.	5,000	Multiaggregation (diameter not reported)
Hildebrandt et al. (2011)	human BM-MSCs	3 to 10	Dynamic (*in situ*) Non-adherent bacterial plate on a rotating platform	Small-scale: Plate on a rotating platform	37°C	75 for 24 h then raised to 85 to 95	nr. 21% ia.	αMEM + 15% FBS + pen/strep	21 days	6 × 10^4^ cells per well	nr.	10000/15000/20000	Diameter 267, 382 and 435 µm
Baraniak & McDevitt, (2012)	Murine BM-MSCs	8 to 10	Static (*ex situ*) Microwells non-adherent plates	Small-scale: Bacterial culture dishes on a rotating platform	37°C	45	nr. 21% ia.	CEM + 10% FBS + 10% HS + 2 mM l-glutamine + pen/strep/ampho	21 days	1.8–6 × 10^6^ cells/ml (static) and 1.500 spheroids in 10 ml (dynamic)	nr.	300/600/1,000	Area 19.3, 24.7, and 7.9 × 10^3^ μm^2^ by day 7
Bhang et al. (2012)	human CB-MSCs	*	Static (*ex situ*) HP	Small-scale: SF	37°C	70	21 and 1%	αMEM + 10% FBS	2 days	1 × 10^4^-2 × 10^6^ cells/ml (static) and 6 × 10^5^ cells/ml (dynamic)	nr.	300/60000	Diameter 200–400 µm
Cho et al. (2012)	human AD-MSCs	>5	Static (*ex situ*) HP	Small-scale: SF	nr. 37°C ia.	70	21 and 1% O_2_.	αMEM + 10% FBS + pen/strep	2 days	1 × 10^6^ cells/ml l (static) and 4.2 × 10^7^ cells/ml (dynamic)	nr.	30,000 app.	nr.
Alimperti et al. (2014)	human BM-MSCs	4	Dynamic (*in situ*) Single cell inoculation in SF	Small-scale: SF	nr. 37°C ia.	80	21% O_2_	Serum-free medium	7 days	1 × 10^5^ cells/ml	nr.	Random	nr.
Bhang et al. (2014)	human AD-MSCs	up to 5	Static (*ex situ*) HP	Small-scale: SF	37°C	45	21 and 1%	αMEM or CRM	4 days	30 µl drople with 1 × 10^6^ cells/ml (static) 6 × 10^5^ cells/ml (dynamic)	nr.	30.000 app.	nr.
Zimmermann and Mcdevitt, (2014)	human BM-MSCs	4	Static (*ex situ*) Microwells in non-adherent plates	Small-scale: Bacterial culture dishes on a rotating platform	37°C	65	nr. 21% O_2_ ia.	Serum-free MesenCult-XF.IFN-y and TNFα were added on day 1	4 days	1.2, 3.0, and 6.0 × 10^6^ cells/ml (formation) and 3 × 10^6^ cells/ml (dynamic)	Yes. IFN-ϒ and TNF-α.	200/500/1,000	nr.
Kwon et al. (2015)	human AD-MSCs	5	Dynamic (*in situ*) Single-cell inoculation in SF	Small-scale: SF	37°C	70	nr. 21% ia.	αMEM + 10% FBS + pen/strep	5 days	1 × 10^6^ cells/ml	nr.	Random	nr.
Li et al. (2015)	human UC-MSCs	1	Dynamic (*in situ*) Single-cell inoculation in dishes on a rocker system	Small-scale: Bacterial culture dishes on a rocker system	37°C	10	nr. 21% ia.	Serum-Free Medium Bao et al. (2013)	9 days	1 × 10^6^ cells/mI	nr.	Random	Diameter of 118 μm at day 9
Santos et al. (2015)	human UC-MSCs	3 to 12	Dynamic (*in situ*) Single-cell inoculation in SF	Small-scale: SF	37°C	80 (formation) 110 (culture)	nr. 21% ia.	MEM +2 mM l-glutamine + 1 g/L glucose + 2.2 g/L sodium bicarbonate + 10% FBS	11 days	1 × 10^6^ cells/ml	nr.	Random	Diameter of 143 μm at day 2 and 309 μm from day 4–11.
Zhang et al. (2015)	human AD-MSCs	5	Dynamic (*in situ*) RWV	Large-scale: RWV	37°C	25	nr. 21% ia.	DMEM/F12 + 10%(v/v) FBS + 1% pen/strep	5 days	1 × 10^6^ cells/ml	nr.	Random	Diameter of 123 μm at day 5
Costa et al. (2017)	human BM-MSCs	4 to 6	Static (*ex situ*) Microwells in non-adherent plates	Small-scale: Ultralow attachment plate on a rotatory orbital shaker	37°C	65	nr. 21% ia.	DMEM + 10% FBS+ 1% antibiotics-antimycotics	7 days	5 × 10^4^ cells/ml	nr.	500	Area of 56400 μm^2^ at day 7
Egger et al. (2017)	human AD-MSCs	2	Dynamic (*in situ*) Single-cell inoculation on STR	Large-scale: STR	37°C	600	21 and 5% O_2_	αMEM + 0.5% gentamycin + 10% human platelet lysate + 1 U/ml heparin	6 days	1 × 10^5^ cells/ml	nr.	Random	nr.
Cha et al. (2018)	human BM-MSCs	2	Static (*ex situ*) Microwells in non-adherent plates	Small-scale: Plates in an orbital shaker	37°C	30	nr. 21% ia.	DMEM +10% FBS or exosome-free FBS + 1% antibiotics-antimycotics	7 days	400 cells per well (formation)	nr.	400 app.	Diameter of 150 µm (post-formation)
He et al. (2019)	rabbit BM-MSCs	5	Dynamic (*in situ*) Single-cell inoculation on SF	Small-scale: SF	nr. 37°C ia	40/45/50	nr. 21% ia.	αMEM + 10% FBS + pen/strep	5 days	2,4 and 8 × 10^5^ cells/ml (at 50 rpm)	nr.	Random	Diameter of 40–60 µm
Allen et al. (2019)	human SyF-MSCs	5	Static (*ex situ*) Microwells in non-adherent plates	Large-scale: STR	37°C	80	nr. 21% ia.	PPRF-msc6 serum-free	12 days	5 × 10^5^ cells/ml	nr.	500/1000/1,500/2000	Diameter of 121, 145, 161 and 181 μm (spheroid formation) and 124 μm on day 1 and increased to 643 μm by day 12 (dynamic culture)
Allen et al. (2019)	human SyF-MSCs	5	Dynamic (*in situ*) Single-cell inoculation in a STR	Large-scale: STR	37°C	80	nr. 21% ia.	PPRF-msc6 serum-free	12 days	5 × 10^5^ cells/ml	nr.	Random	Diameter of 100 μm at day 6, 125 μm on day 8, 137μm on day 10, and 153 μm on day 12.
Miranda et al. (2019)	human UC-MSCs	3 to 12	Dynamic (*in situ*) Single-cell inoculation in SF	Small-scale: SF	37°C	80 (formation) and 110 (culture)	nr. 21% ia.	αMEM + 15% FBS	7 days	1 × 10^6^ cells/ml (dynamic)	nr.	Random	Diameter of 195.48 µm from day 5–7 of culture
Niibe et al. (2020)	human BM-MSCs	4 to 7	Dynamic (*in situ*) Single-cell inoculation in SF	Small-scale: SF	37°C	85–95	nr. 21% ia.	αMEM, GlutaMAX-I, 10% FBS, 1% p/s, 10 mM hepes, 20 ng/ml FGF2.	1–2 months	5 × 10^5^–5 × 10^6^ cells/ml	nr.	Random	Feret’s diameter 5 × 10^5^: 699 µm (1) 757 µm (2) 5 × 10^6^: 782 µm (1) 833 µm (2)
Niibe et al. (2020)	human BM-MSCs	17 to 20	Dynamic (*in situ*) Single-cell inoculation in SF	Small-scale: SF	37°C	85–95	nr. 21% ia.	αMEM, GlutaMAX-I, 10% FBS, 1% p/s, 10 mM hepes, 20 ng/ml FGF2.	1–2 months	5 × 10^5^–5 × 10^6^ cells/ml	nr.	Random	Feret’s diameter 5 × 10^5^: 534 µm (1) 734 µm (2), 5 × 10^6^: 760 µm (1) 1,158 µm (2)

**TABLE 2:** Comparison of different studies reporting static and dynamic methods of spheroid formation and their impact on the CM profile.

References	MSCs source	Formation method	Viability and proliferation	CM characterization and secretion of paracrine factors	Other results
Frith et al. (2010)	human BM-MSCs	Static (*ex situ*) Microwells in non-adherent plates	Most of the cells were viable, with no differences between cells on the periphery and those in the centre; 3.9, 4.3 and 3.5% of cycling cells for monolayer, SF and RWV culture.	CM obtained from MSCs cultured in monolayer or stirring flasks (3D) was used to treat both MSCs and other cells from the bone marrow microenvironment and viability was determined by MTT assay. The results showed no difference in the viability of primary MSCs, C3H101T1/2 or human umbilical vein endothelial cells cultured in 2D and 3D CM. However, the viability of the human osteosarcoma cell line Saos-2 was significantly decreased in cells from 3D versus 2D CM. No secretion of paracrine factors reported.	Increased osteogenic and adipogenic differentiation potential of MSCs spheroids compared with MSCs cultured at 2D.
Bhang et al. (2011)	human AD-MSCs	Dynamic (*in situ*) Single-cell inoculation in SF	Cell adhesion and migration were preserved.	Increased secretion of angiogenic factors (VEGF, HGF, FGF2 and CXCL12) from hADSC cultured as spheroids versus hADSC cultured at monolayer. HGF: 750 vs 200 [pg per 10^4^ cells], VEGF: 780 vs 400 [pg per 10^4^ cells] and FGF2: 500 vs 250 [pg per 10^4^ cells].	Transplantation of spheroids promoted angiogenesis in mouse ischaemic limb tissue.
Hildebrandt et al. (2011)	human BM-MSCs	Dynamic (*in situ*) Non-adherent bacterial plate on a rotating platform	Very low viability estimated by PI/FDA staining	No secretion of paracrine factors reported.	Very heterogeneous spheroids in size and number. Aggregates form larger amorphous masses, with low viability after 1 week.
Hildebrandt et al. (2011)	human BM-MSCs	Static (*ex situ*) HP	Small aggregates are viable, estimated by PI/FDA staining	No secretion of paracrine factors reported.	Increased multiaggregation compared with spheroids formed by HP.
Hildebrandt et al. (2011)	human BM-MSCs	Dynamic (*in situ*) Non-adherent bacterial plate on a rotating platform	High viability estimated by PI/FDA staining. However, a clear tendency towards decreased viability at day one by WST-1 assay.	No secretion of paracrine factors reported.	Increased efficiency of formation and more controlled size compared with spheroids cultured under dynamic conditions, at lower initial cell density.
Baraniak & McDevitt, (2012)	Murine BM-MSCs	Static (*ex situ*) Microwells non-adherent plates	Absence of necrotic core. BrdU staining confirmed the retention of MSC proliferative capacity after dynamic culture.	No secretion of paracrine factors reported.	Increased adipogenic and osteogenic potential of cells recovered from 3D cultures. Maintenance of MSCs plasticity following 3D culture.
Bhang et al. (2012)	human CB-MSCs	Static (*ex situ*) HP	MTT assay showed that cell viability is higher in spheroids than in 2D cultures. No proliferation was reported.	Increased secretion of HGF, VEGF and FGF2 from hADSC cultured as spheroids versus hADSC cultured at monolayer. Values not reported.	Increased expression of *Bcl-2* in spheroids cultured under hypoxia compared with that in 2D cultures under normoxia and hypoxia or 3D cultures under normoxia
Cho et al. (2012)	human AD-MSCs	Static (*ex situ*) HP	nr.	Increased secretion of TGFβ1 and VEGF of CM obtained from 3D cultures compared with baseline culture medium (αMEM). VEGF: 1,015.17 ± 170.97 [pg/ml], TGFβ1: 14.33 ± 6.71 [pg/ml]. *Increased in vitro* pro-angiogenic effects of CM obtained from 3 days cultures confirmed by a tube-formation assay. The continuous infusion of CM obtained from 3D cultures induced significantly better functional and structural recovery after stroke, reducing the infarction volume and maintained motor function in an ischemic stroke model.	-
Alimperti et al. (2014)	human BM-MSCs	Dynamic (*in situ*) Single cell inoculation in SF	Spheroids showed a 80% of viability until day 5. Cells were quantified with an automatic cell counter Vi-CELL, which measures viable cell density, viability and average cell size.	No secretion of paracrine factors reported.	High levels (>99%) of MSC surface markers. Increased trilineage differentiation potential for spheroids cultured in serum-free medium.
Bhang et al. (2014)	human AD-MSCs	Static (*ex situ*) HP	3D spheroid culture system was able to support the growth of cells at a density app. 4 times higher than that observed for 2D cultures, for both media.	CM obtained from spheroids culture had a significantly higher concentration of angiogenic factors than the monolayer culture CM (VEGF, FGF2, HGF, and CXCL12 in the αMEM (serum+) spheroid culture CM was 14.4 ± 0.4, 13.2 ± 2.2, 13.3 ± 2.3, and 16.6 ± 2.9 [ng/ml], respectively). *In vitro* and *in vivo* antiapoptotic effect, was observed in an ischemic hindlimbs model. In addition, *in vivo* angiogenic effect of CRM-bases spheroid CM. and an improved blood perfusion in the ischemic limbs.	Spheroids cultured in CRM (without serum) supported culture at a significantly higher maximal cell density compared with the monolayer culture supplemented with serum (×10^5^ cells/ml; 7.0 ± 0.8 versus 3.1 ± 0.5). Serum deprivation caused CASP3 pathway activation and increased TP53 mRNA expression, regardless of the type of medium or culture system used.
Zimmermann and Mcdevitt, (2014)	human BM-MSCs	Static (*ex situ*) Microwells in non-adherent plates	↑ no. of spheroids in Mesencult-XF In FBS medium, no change in the total number of cells in spheroid cultures occurred after 4 days, indicating no significant expansion in MSCs over the culture period. A 1.9, 2.0 and 2.9-fold change in the total number of cells was observed in 200-cell, 500-cell and 1000-cell spheroid culture in MesenCult-XF medium after 4 days.	Increased secretion of PGE2, TGFβ1, and IL6 from spheroids hMSC, compared with human MSCs cultured. Increased secretion of IL6 in spheroids cultured in MeseCult-XF medium compared with cells grown in 2D culture, which did not secrete detectable levels of IL6. Increased secretion of immunomodulatory factors by 500-cell spheroids	-
Kwon et al. (2015)	human AD-MSCs	Dynamic (*in situ*) Single-cell inoculation in SF	nr.	Monolayer cultured hADSCs in αMEM medium without supplemental serum or supplements, secreted **VEGF** (0.56 ± 0.22 ng/ml), **FGF2** (0.51 ± 0.06 ng/ml), **HGF** (0.55 ± 0.08 ng/ml), and CXCL12 (0.085 ± 0.07 ng/ml). When hADSCs were cultured in spheroid culture, significant increases in VEGF (12.3 ± 2.4 ng/ml), FGF2 (11.0 ± 1.7 ng/ml), HGF (10.8 ± 3.6 ng/ml), and SDF-1a (12.5 ± 3.8 ng/ml) were observed. The concentration of growth factor per cell in spheroid culture CM was approximately 20-fold higher for VEGF, FGF2, and HGF and 145-fold higher for CXCL12 as compared with the monolayer culture CM.	Increased cell density in 3D (10.6 × 10^5^ cells/ml) vs. 2D culture (2.95 × 10^5^ cells/ml).
Li et al. (2015)	human UC-MSCs	Dynamic (*in situ*) Single-cell inoculation in dishes on a rocker system	Over 95% at day 9. Ki-67 staining showed that the cells retained their ability to proliferate.	No secretion of paracrine factors reported.	Increased expression of *Oct4, Nanog, Sox2* and *Rex1*, with 4.3, 3.9, 6.2 and 3.2-fold increases vs. 2D culture.
Santos et al. (2015)	human UC-MSCs	Dynamic (*in situ*) Single-cell inoculation in SF	Absence of necrotic core at day 11. Ki67 staining showed the presence of proliferating cells. However, Ki67 positive cells comprised only a small fraction of cells, indicating that only a low fraction (<5%) of cells were actively proliferating in spheroids.	Increased secretion of HGF, TGFβ1, FGF2, IL6, and GCSF in CM obtained from 3D cultures than in CM from 2D cultures. Most impressively, VEGFA, which was only residually secreted in 2D cultures, was highly secreted by MSCs under 3D conditions (80-fold higher than CM obtained from 2D). The results strongly suggested an improved paracrine effect of CM obtained from 3D cultures onto fibroblast-mediated ECM synthesis, angiogenesis and vasculogenesis, essential for the granulation tissue formation and remodelling stages of wound healing.	From day 6 onwards, the population of 3D spheroid-dissociated cells showed a decrease in CD105 and C90 expression levels that restored once spheroids were plated back. MSCs grown in 3D cultures were app. 30% smaller in size when compared to cells grown in 2D. In addition, MSCs retained the ability to adhere and proliferate on plastic surface and tridifferentiation potential.
Zhang et al. (2015)	human AD-MSCs	Dynamic (*in situ*) RWV	Most of the cells were viable. Absence of necrotic centre. Spheroid-derived ADSCs exhibited significantly stronger proliferative ability than cells grown in 2D culture at later time points.	In addition, the concentration of growth factors per cell in spheroid culture CM was 20-fold higher than that in 2D culture CM for VEGF, FGF2, and HGF and 145-fold higher for CXCL12.	Increased expression levels of Oct4, Nanog, Sox2, and Rex1 compared with those in 2D culture.
Costa et al. (2017)	human BM-MSCs	Static (*ex situ*) Microwells in non-adherent plates	Similar cell viability values were found for both monolayers and spheroids.	Increased secretion of HGF and VEGF. .CM obtained from 3D cultures exhibited higher closure of the wounded area 8 h after (app. 40%) relatively to monolayer-derived CM (app. 27%) in an *in vitro* scratch wound healing assay.	Spheroids exhibited increased resistance to oxidative stress compared t single MSCs. Increased expression level of *TSG6*.
Egger et al. (2017)	human AD-MSCs	Dynamic (*in situ*) Single-cell inoculation on STR	78.5% (normoxic) and 86% (hypoxic) viability; 1.85 - fold (normoxic) and 2.23 -fold (hypoxic) cell expansion.	No secretion of paracrine factors reported.	Surface markers of cells cultivated under normoxic and hypoxic conditions were comparable and met the minimal criteria of MSCs.Increased adipogenic and chondrogenic differentiation under 3D hypoxic culture vs. 3D normoxic culture.Decreased osteogenic differentiation under 3D hypoxic culture vs. 3D normoxic culture.Glucose consumption (0.85 ± 0.1 mmol) and lactate production (1.69 ± 0.11 mmol) were significantly lower in normoxic conditions compared to hypoxic conditions, where glucose consumption was 1.09 ± 0.02 mmol and lactate production 2.05 ± 0.09 mmol.
Cha et al. (2018)	human BM-MSCs	Static (*ex situ*) Microwells in non-adherent plates	Live/Dead assay showed that most cells in the spheroids were viable during the culture period. No changes in spheroid numbers.	Increased production of MV. Highest enrichment of hMSC-derived MVs was found in dynamic 3D cultures, which was approximately 100-fold higher than in the 2D control containing only a few secreted MVs.	Upon formation of hMSC-spheroids at day 1, *GDF15* and *TGFB3* were upregulated by approximately 40-fold compared to the 2D control, whereas *BMP4* was downregulated by approximately 60-fold. At day 7, *IL1B*, *BDNF*, and *BMP2* were upregulated by over 30-fold while *COL1A1* was downregulated by approximately 50-fold, compared to the early stage on day 1.
He et al. (2019)	rabbit BM-MSCs	Dynamic (*in situ*) Single-cell inoculation on SF	nr.	No secretion of paracrine factors reported.	After 24 h of culture, approximately 80% of rMSCs were incorporated into cellular aggregates. Faster aggregation at a lower agitation rate and a higher cell inoculation density.
Allen et al. (2019)	human SyF-MSCs	Static (*ex situ*) Microwells in non-adherent plates	Proliferation ceased at day 6.	No secretion of paracrine factors reported.	Increased collagen production in spheroids. Highly variable size of spheroids in dynamic culture.
Allen et al. (2019)	human SyF-MSCs	Dynamic (*in situ*) Single-cell inoculation in a STR	Proliferation ceased at day 6.	No secretion of paracrine factors reported.	Single-cell inoculation yields a more uniform population of smaller aggregates after eight days of culture. Single-cell and preformed spheroid inoculation achieved similar fold changes in cell numbers and SGAG.
Miranda et al. (2019)	human UC-MSCs	Dynamic (*in situ*) Single-cell inoculation in SF	Spheroids were viable until day 7 given haematoxylin and eosin staining images.	Increased secretion of anti-inflammatory cytokines such as IL10 and LIF, as well trophic factors involved in different mechanisms leading to tissue regeneration, mainly PDGFB, FGF2. CCL1 SCF and GMCSF in CM obtained from 3D cultures. CM derived from 3D dynamic cultures promoted a 1.5-fold increase in chondrocyte migration capacity 24 h post-scratch in an *in vitro* healing assay, when compared to CM obtained from 2D cultures Also, results showed that CM obtained from 3D cultures has a clearly superior capacity for both, avoiding and ameliorating adjuvant induced arthritis (AIA) manifestations *in vivo* when compared to CM obtained from 2D cultures or even MSCs. CM treatment was able to both prevent a reverent all major signs of AIA, including complete avoidance of necrotic foci around joints, acute and chronic inflammation, joint deformity and secondary infection.	-
Niibe et al. (2020)	human BM-MSCs	Dynamic (*in situ*) Single-cell inoculation in SF	Live/dead staining after 4 weeks dynamic 3D culture showed that most cells on the spheroids surface and outer layer were alive.	No secretion of paracrine factors reported.	Decreased expression level of *Ki67*, known as a market of cell proliferation, suggest that the dynamic 3D culture mimicked a state of quiescence or stopped the MSCs cell cycle.
Niibe et al. (2020)	human BM-MSCs	Dynamic (*in situ*) Single-cell inoculation in SF	Live/dead staining after 4 weeks dynamic 3D culture showed that most cells on the spheroids surface and outer layer were alive.	No secretion of paracrine factors reported.	Increased expression level of CD27 in spheroids.

## 5 Key Variables in Dynamic Culture: Impact on Spheroid Formation and Conditioned Medium Production

The success of an MSC bioprocess, in terms of expansion, differentiation, and secretion of bioactive factors, will mainly depend on the ability to control critical variables: concentration of nutrients and metabolites, the composition of the culture medium, temperature, pH, oxygen, and shear stress.

### 5.1 Initial cell Density (Inoculum): Spheroidal diameter and its Implications

**FIGURE 2:**
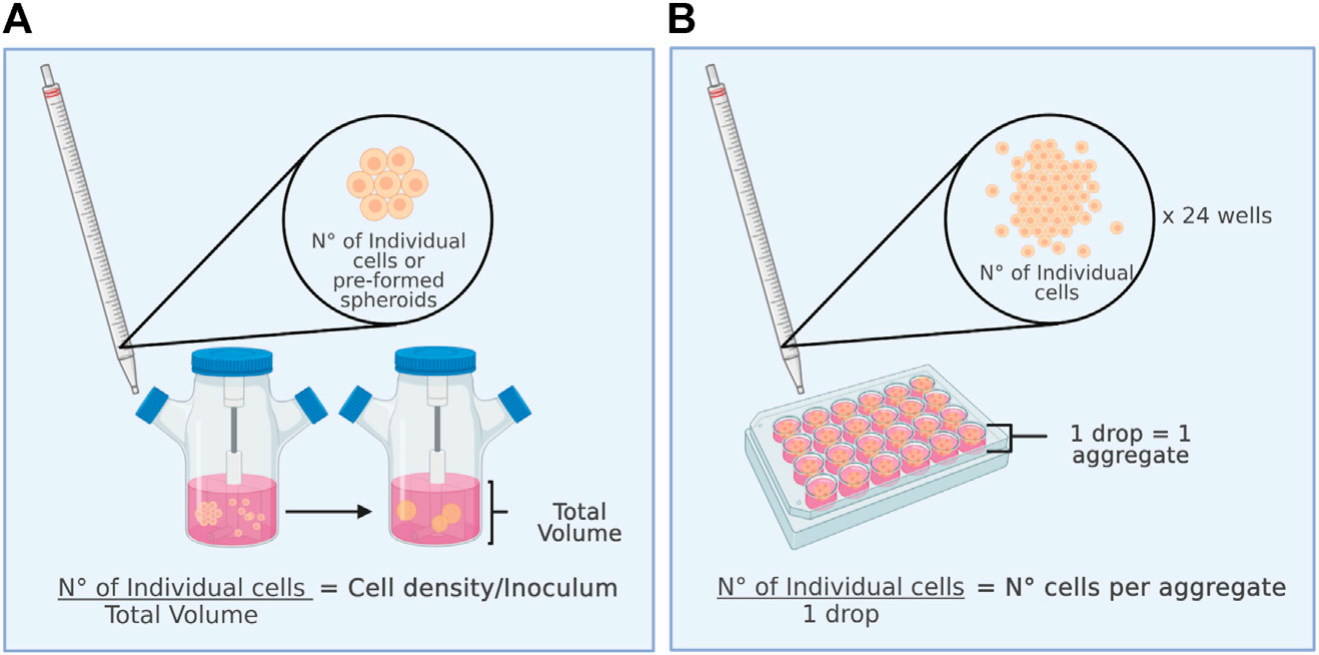
The inoculum is a key parameter for the dynamic culture of spheroids. **(A)** (Left figure) Initial cell density and **(B)** number of cells per aggregate.

In most studies on 3D MSCs culture, the initial inoculum density (Figure 2A) or the number of cells per aggregate (Figure 2B) is a relevant parameter to consider. When spheroid formation is performed *in situ*, the inoculum density directly affects the number of cells to be aggregated per spheroid, influences their diameter and size, and can impact their metabolism, viability, and cell proliferation capacity. In contrast, the number of cells per aggregate is considered when spheroids are formed by an *ex situ* static culture method. *Ex situ* static culture makes it possible to directly control the number of cells that will form an aggregate. Later, to characterize the aggregates post-dynamic culture, it is necessary to disaggregate them to obtain single cells for counting. Figure 2 shows the differences between the initial cell density and the number of cells per aggregate for better understanding.

The inoculum density in dynamic MSC spheroid cultures varies from 1 × 10^4^ to 1 × 10^6^ cells/ml (Frith, Thomson et al., 2010; Bhang, Lee et al., 2014; Kwon, Bhang et al., 2015; Santos, Camoes et al., 2015; Zhang, Liu et al., 2015; Egger 2017; Allen, Matyas et al., 2019). Preformed spheroids were cultured for 7 days in stirring flasks and RWV bioreactors at a low inoculum density of 20,000 cells/ml with 15% FBS and 21% O_2_, yielding average diameters of 98.7 and 31.7 μm, respectively (Frith, Thomson et al., 2010). In dynamic culture at an intermediate density, with 6 × 10^5^ cells/ml in 10% FBS with 1% O_2_ and a shaking speed of 70 rpm, aggregates are formed in 24 h with diameters varying between 100 and 350 μm, with a higher frequency between 200 and 250 μm (Bhang, Cho et al., 2011). In a RWV bioreactor at 25 rpm with a high inoculum of 1 × 10^6^ cells/ml, cells aggregate into spheroids within 24 h. The size of the spheroids increases gradually on days 1 and 5 (77 ± 25 μm to 124 ± 26 μm), and the number of suspended cells continues to decrease. Most of the cells were viable within the spheroids from RWV culture, and no necrotic centres were observed (Zhang, Liu et al., 2015). For process optimization, it is crucial to consider that the formation of large agglomerates that increase their volume during culture is a recurrent problem during spheroid suspension culture. A follow-up that is not usually done but that would be convenient to carry out is to monitor the formation process of the 3D structures. Recently, a strategy was developed to monitor the spheroid formation process of human-induced pluripotent stem cells in custom-made STR. This innovative platform integrates a monitored culture into an automated incubator, including *in situ* microscopic imaging to visualize hiPSC aggregation in real time. In this way, a significant amount of data can be obtained for detailed morphological characterization of the aggregates (1,000 aggregates per minute), in contrast to a manual sampling procedure, where considerably fewer counts are performed (Schwedhelm, Zdzieblo et al., 2019). These types of systems, however, have not been used in self-aggregation culture of MSCs. Using these methodologies to implement spheroid suspension culture would allow greater robustness and reproducibility, keeping culture manipulation and, therefore, the risk of contamination of the product of interest with microorganisms to a minimum.

### 5.2 Shear Stress

The hydrodynamic conditions generated in dynamic culture are essential to maintain a homogeneous cell suspension. Mechanical agitation causes the transfer of energy from the impeller to the medium, generating high-turbulence zones. This turbulence generates hydrodynamic stress called shear stress. When localized at the spheroid-fluid interface, shear stress can induce a cellular response, triggering molecular processes that influence the multipotentiality and differentiation capacity of the cells (Dong, Gu et al., 2009; Yourek, McCormick et al., 2010; Borys, Le et al., 2019). This shows that MSCs in culture can respond and adapt to shear stress as under physiological conditions. In fact, in their *in vivo* niche, cells from mesenchymal tissues are constantly subjected to mechanical forces, so they can respond rapidly to these stimuli (Kelly and Jacobs 2010). Furthermore, *in vitro* assays have shown that MSCs undergo morphological changes and cytoskeletal rearrangement when exposed to 0.25–1 Pa shear stress for 24 h (Kim, Heo et al., 2011).

In spheroid culture, it is essential to consider that an optimal range of shear stress exists. The lower the shear stress is, the greater the tendency to generate superaggregates, while the higher the shear stress is, the greater the probability of cell death. For the case of endothelial and embryonic cell multiaggregates in dynamic culture at 80–120 rpm in stirring flasks (100 ml volume) optimal shear stress values (maximal cell density point) of 0.2–0.6 Pa have been reported, achieving a 31-fold expansion and a peak viable density reaching 1 × 10^6^ cells/ml (Cormier et al., 2006). In 3D culture of MSCs, maximum shear stress between 0.3 and 0.6 Pa have been observed in stirring flasks at 40 rpm (100 ml volume, equipped with 90° paddles, radial flow and magnetic stir bar), meanwhile 0.08 Pa average of shear stress value have been reported at 60 rpm(Mizukami, Fernandes-Platzgummer et al., 2016), in a 2.5 L STR (equipped with a tree-blade pitched impeller, 45° angles, radian and axial flow), with an 800 ml working volume and achieving a maximum cell density of 1.4 × 10^5^ cells/ml. The MSCs cultured in this case were associated with a microcarrier, so the response differs from what might be observed in a support-free self-aggregation culture. However, the results show significant differences between maximum shear stress at which MSCs were exposed in a stirring flask compared with MSCs cultured in a STR with proper agitation system. For spheroids, cultures have been reported with speeds of 80–110 rpm in 125 ml stirring flasks (Santos, Camoes et al., 2015), which allows the maintenance of an aggregate diameter of less than 350 µm without observing damage to or detrimental effects on MSCs. Even at a stirring speed of 600 rpm in a STR (130 ml volume, impeller type not described), and a shear stress of 0.05–0.35 Pa, MSCs in spheroids maintain their marker expression, with a viability of 78.5% (Egger, Schwedhelm et al., 2017). According to these results, it is possible to modulate the shear stress level generated in a dynamic system with a sufficient magnitude to produce spheroids of adequate size while maintaining the viability and properties of MSCs. In addition, future work should focus on the design and geometry of the impellers, which according to this review has substantial impact on the shear stress to which the MSCs will be exposed.

### 5.3 Oxygen Tension

For dynamic cultures, one key variable that needs to be considered is the oxygen supply. If cells use oxygen faster than it is being supplied, then the dissolved oxygen level will decrease to a point where the culture may not support cell growth. Traditionally, the expansion and culture of MSCs have been performed in conventional incubators at 37°C and 5% CO_2_, with dissolved oxygen in equilibrium to atmospheric O_2_ levels, which represents 21% oxygen. Inside the incubator, the volume fraction of oxygen reaches 18.6% because of the addition of partial pressures of CO_2_ and water vapor (Pavlacky and Polak 2020). Thus, 18.6% O_2_ is widely considered standard or *in vitro* normoxia.

The level of O_2_ varies markedly among different tissues (Place, Domann et al., 2017). Tissue normoxia in bone marrow is 7%, while in avascular environment, it is between 1% and 3% (Mohyeldin, Garzon-Muvdi et al., 2010). These differences suggest that depending on the origin of MSCs, their O_2_ requirements may vary and O_2_ levels may have different effects on their proliferation and functionality. In this regard, it has been reported that MSCs culture under *in vitro* normoxic conditions induces severe DNA damage, contributing to senescence, loss of cell viability and significant decreases in the levels of secreted factors (Busuttil, Rubio et al., 2003; Jin, Kato et al., 2010). Hypoxia does not have a strict definition in terms of oxygen tension and is dependent on cell type and physiological conditions. In this work, we defined hypoxia as a controlled atmosphere at 1–5% partial pressure of O_2_ that promotes genetic stability, proliferation, viability and cell differentiation of MSCs (Greyson, Zhao et al., 2006, Estrada, Albo et al., 2012; Valorani, Montelatici et al., 2012) and the secretion of bioactive factors. Increases in VEGF and FGF2 levels in CM of up to 2.8-fold have been reported when MSCs are grown at 2% O_2_ in 2D cultures. (Valorani, Montelatici et al., 2012). Likewise, it has been observed that the combined effect of hypoxia and the absence of serum on MSCs increases the secretion of factors such as EGF, TGF-α, and IL10 (Russell et al., 2017). Additionally, in experimental models of brain injury, MSCs 2D-cultured at 4% O_2_ improved motor and cognitive functions (Chang, Chio et al., 2013). All these data suggest that hypoxia directly enhances the secretion of factors.

Different oxygen levels have a more significant effect on MSCs 3D culture than 2D culture. During the formation of multiaggregates, molecular gradients of medium components, including O_2_, are created. As cells in the outer layers of the aggregates consume O_2_, cells in the inner regions of the aggregate face a hypoxic environment. The hypoxia level in the centre of the aggregate will depend on the aggregate’s diameter, and it has been postulated that the larger the diameter is, the more necrotic the core becomes (Sart, Tsai et al., 2014). However, spheroids containing 6 × 10^4^ cells per spheroid (350 μm diameter) exhibit a less than 10% decrease in OT between the outer layer of cells and the inner core (Murphy, Whitehead et al., 2017). The same study showed that a statistically significant hypoxic core was observed beginning from a size of 2.5 × 10^5^ cells per spheroid. Additionally, MSCs derived from 3D dynamic cultures seem to be more adaptable and resistant to hypoxia than MSCs cultured at monolayer Indeed, exposure to 1% O_2_ for 3 days and subsequent formation of MSC spheroids increases VEGF secretion and accelerates bone formation *in vivo* (Ho, Hung et al., 2018). MSC spheroids cultured at 2% O_2_ show increased production of ECM components, including laminin, elastin, collagen I and fibronectin, and increased secretion of VEGF and FGF2 (Shearier, Xing et al., 2016) compared with those cultured at 20% O_2_. Higher levels of ECM in spheroids were associated with increased formation of multiaggregates (Indovina, Rainaldi et al., 2008; Inamori, Mizumoto et al., 2009) and enhanced apoptosis resistance both in cell culture and after implantation *in vivo* (Santini, Rainaldi et al., 2000; Zahir and Weaver 2004).

All these data suggest that adequate oxygen tension in combination with 3D dynamic culture can considerably improve the secretion of bioactive factors, directly affecting the composition and quality of the CM. Therefore, the study of the oxygen tension level as an operational variable is of great interest. In bioreactors, oxygen is typically supplied to the medium by sparging swarms of air bubbles underneath the impeller. The action of the impeller then disperses gas throughout the vessel. In this way, the system ensures a homogeneous distribution of cells, nutrients, and gases. To date, there has only been one published work on STR culture of MSCs. The results have shown that MSC spheroids cultured in an STR with 5% hypoxia for 6 days exhibited a 10% increase in aggregate viability without altered expression of MSC markers post-culture, slightly increasing differentiation into adipocytes and chondrocytes (Egger, Schwedhelm et al., 2017). However, no data related to CM were reported.

### 5.4 Temperature

Temperature is one of the operational conditions that significantly impacts cell growth, yet it is one of the least studied variables in the context of MSCs culture. It has a substantial effect at the metabolic level, directly affecting reaction rates. It can also change various cellular behaviours, especially protein expression. In addition, it is an operational variable that can be easily measured, controlled, and modified.

Considering mammalian body temperature, the temperature of cell cultures has traditionally been set at 37°C. However, in recent years, many investigations involving the production of recombinant proteins in mammalian cells have determined that cell culture at subphysiological temperatures (30–33°C), referred to as mild hypothermia, represents a powerful strategy to maximize the productivity of biosystems (Yoon, Hong et al., 2006; Sunley, Tharmalingam et al., 2008; Becerra et al., 2012; Vergara, Becerra et al., 2014; Torres, Zuniga et al., 2018). The mechanisms proposed to explain the positive effect of mild hypothermia on crop-specific productivity include increased abundance of mRNA stabilizing proteins, increased capacity of the endoplasmic reticulum for protein folding and processing, decreased apoptosis, reorganization of the cytoskeleton, decreased carbon metabolism, and reduced oxidative stress, among others (Fogolin, Wagner et al., 2004; Becerra et al., 2012; Bedoya-López, Estrada et al., 2016). In addition, in the clinical setting, moderate hypothermia exerts a neuroprotective effect by reducing ischaemic and traumatic brain damage (Ahmed, Bullock et al., 2016), as it inhibits neuronal apoptosis (Luo and Pan 2015). This increased tolerance to an adverse environment has also been reported in MSCs, where moderate hypothermia of 33°C for 24 h decreases apoptosis induced by 1% O_2_ (Liu, Ren et al., 2017). Culturing MSCs at 32°C for 7 days while reducing the proliferation rate does not affect their expression of phenotypic markers, does not alter their osteogenic differentiation potential, and increases their antioxidant activity even at late passages (Stolzing and Scutt 2006). Compared with the 37°C differentiation protocol, induction of adipogenic differentiation for 9 days at 32°C did not affect cell viability or increase metabolic activity or adipogenesis (Velickovic, Lugo Leija et al., 2018). Despite these findings, to date, there is no report on how moderate hypothermia affects the secretome of MSCs cultured in 2D or 3D, let alone whether it affects spheroid formation or differentiation potential.

The relationship between hyperthermia (39–42.5°C) and MSCs has also not been studied in depth, despite the importance of the febrile response that characterizes systemic inflammation and infectious diseases. Studies in human immune cells showed that periods of mild hyperthermia (39 and 40°C) increase the expression of IL10, TNF-α and various chemokines that promote the migration capacity of dendritic cells and the activation of CD8^+^ T lymphocytes (Knippertz, Stein et al., 2011), in addition to inducing a pattern of gene expression associated with posttranscriptional modifications, protein folding and chemotaxis (Liso, Castellani et al., 2017). In cardiomyocytes, thermal shock at 39°C induced an increase in the expression of Hsp70, one of the main cellular stress-related proteins, associated with a protective effect against oxidative stress *in vitro* and *in vivo*. Exposure of BM-MSCs 2D-cultured to a temperature of 42°Cat time intervals of 15–60 min induce the expression of Hsp70 and Hsp27 (Moloney, Hoban et al., 2012), two proteins associated with the regulation of apoptosis signal transduction (Takayama, Reed et al., 2003). It has even been observed that administration of recombinant Hsp70 to aged murine MSC 2D-cultures (passage 15), coupled with heat shock at 42°C for 5 min, induces a kind of cellular “rejuvenation” that increases proliferation, with an increase of up to a 4-fold in the proliferation of untreated cells at an optimal concentration of Hsp70 (Andreeva, Zatsepina et al., 2016). The most recent study on this topic showed that when coculturing human MSCs with THP1 monocytes at 38.5°C for 1 h (in monolayer), there is a significant increase in IL10 secretion and a decrease in TNF-α levels, associated with an increase in the immunosuppressive potential of MSCs (McClain-Caldwell, Vitale-Cross et al., 2018).

Overall, these results show that changes in MSCs culture temperature could positively affect the quantity and quality of the obtained CM, offering the possibility of regulating the CM profile by modifying a low-cost operational variable. Thus, it is of interest to evaluate different temperatures and their impact on CM, mainly through the dynamic culture of spheroids in bioreactors, where it is possible to measure and control the temperature of the medium with higher homogeneity.

### 5.5 Medium Composition, the Absence of Fetal Bovine Serum and the Use of Human Platelet Lysate

The formulation of the culture medium, particularly the presence or absence of serum, is another factor that can have a substantial impact on MSCs growth when transitioning from static to dynamic culture. Along with growing interest in the study of stem cells and the development of cell therapy as a viable alternative for treating different diseases, in recent years, interest has also increased in the development of serum-free media for the isolation and expansion of MSCs. The principal aims are to avoid the risk of immunological reactions and transmission of contaminants and decrease batch-to-batch variability. From the perspective of MSCs production and cell-free products, serum and supplements in the culture medium directly interfere with the detection and analysis of proteins secreted during culture. This interference occurs because most of the extracellular components are secreted at low concentrations (ng or pg) and can be masked by the added components. Therefore, CM should preferably be obtained from cultures grown under serum-free conditions for at least 24–48 h (Kumar, Kandoi et al., 2019).

The dynamic culture of serum-free MSC spheroids represents a significant challenge since serum provides nutrition and a protective effect against hydrodynamic stress (Fernandes-Platzgummer, Diogo et al., 2011). Although the mechanism of action is still unclear, studies have suggested that the protective role of serum has both a physical component, involving changes in the rheology of the medium, and a biological component, related to the integrity of the actin microfilaments of the cytoskeleton, the fluidity of the plastic cell membrane and the capacity of the cells to maintain an active state of energy metabolism (Papoutsakis 1991; Ramirez and Mutharasan 1992). An alternative to serum-free media is the use of human platelet lysate (hPL). hPL is rich in cytokines and growth factors, and studies have shown that its use improves the expansion of hMSCs without the need to administer additional growth factors (Lawson, Kehoe et al., 2017). hPL is produced under institutional standard operating procedures regulated by the FDA and EMA, and it is a safe alternative medium for MSCs production. hPL can sustain the growth of MSCs without affecting their immunophenotype or functional properties. Recent reports have shown that hPL can either maintain or diminish the immunosuppressive properties of MSCs in 2D cultures (Menard, Pacelli et al., 2013; Oikonomopoulos, van Deen et al., 2015). Lately, a dynamic culture of MSC spheroids with 10% hPL and 600 rpm agitation has been reported, without deterioration or loss of viability, which could be related to the ability of this type of supplement to protect against shear stress (Egger, Schwedhelm et al., 2017). Although the obtained results are promising, the use of hPL also interferes with subsequent analysis of the CM, given that it inherently contains growth factors.

Evidence indicates that the dynamic culture of spheroids under serum-free conditions is feasible and shows promise for the development of strategies focused on the production of CM and cell-free products. However, there have been few studies carried out under these conditions, so it is necessary to expand research on this topic to generate new and better production strategies that follow good manufacturing practices.

### 5.6 Priming

As described above, there are key operational variables involved in the expansion of MSCs under dynamic conditions that influence the secretome profile. However, to achieve optimal paracrine function, it has been suggested that MSCs must undergo a preconditioning process that is usually called priming (Doorn et al., 2012). Oxygen tension, temperature, shear stress and 3D culture configuration are physical factors that, under certain conditions, are used as priming factors. However, biochemical stimulation of MSCs with cytokines, chemokines, blood activation products, Toll-like receptor (TLR) ligation and other approaches is the most commonly used method to precondition MSCs for the production of biomolecules with therapeutic potential (Miceli, Bulati et al., 2021). If the therapeutic application of MSCs is based on their immunomodulatory properties, this *priming* can be more accurately described as “*licensing*,” which implies activation of MSCs (by proinflammatory cytokines or pathogen challenge), the prevalence of *priming* stimuli and the timing of MSCs engagement for the activation of immune effector cells (Krampera 2011; Najar, Bouhtit et al., 2019).

Interferon-γ (IFN-γ) has been the most extensively investigated factor for priming MSCs. Indeed, the ISCT recommends IFN-γ, alone or combined with TNF-α, as a standard priming method for evaluating the immunosuppressive capacity of MSCs *in vitro* (Krampera, Galipeau et al., 2013). In human BM-MSC spheroids generated by forced aggregation, IFN-γ licensing induces strong expression of the immunomodulatory factor indoleamine 2,3-dioxygenase (IDO), which correlates with the ability of MSCs to suppress the T-cell response (Zimmermann, Hettiaratchi et al., 2017). After incubation of MSC spheroids with IFN-γ, IDO levels decreased rapidly. This temporary effect was improved by including heparin microparticles loaded with IFN-γ within the spheroid, resulting in sustained IDO expression that in turn induces sustained MSCs suppression of CD3/CD28-activated T cells *in vitro* (Zimmermann, Hettiaratchi et al., 2017). Additionally, human BM-MSC spheroids generated on low-attachment surfaces have been primed with interleukin-1 (IL-1), resulting in increased secretion of the pro-trophic molecule granulocyte-colony stimulating factor (GCSF) as well as the tissue remodelling proteins MMP13 and TIMP1 (Redondo-Castro, Cunningham et al., 2018). Another static cultivation method used a fibroblast growth factor 2 (FGF2)-immobilized matrix to produce a priming effect during human AD-MSC spheroids formation. Immobilized FGF2 was able to induce strong IL8 expression, improving the angiogenic potential of MSCs *in vitro* and *in vivo*, likely through secretory factors (Choi, Choi et al., 2021). Human AD-MSCs have also been primed with the extracellular matrix protein matrilin-3 before the static formation of spheroids; the results showed that compared to unprimed spheroids, primed spheroids exhibited higher secretion of different cytokines (TGFβ1, TGFβ2, IL10, GCSF and MMP1) and suppression of the acute phase of disc degeneration after implantation in a rabbit model (Muttigi et al., 2020).

MSC spheroids priming has been investigated mostly using static techniques. To our knowledge, only one work evaluated licensing with TNF-α and IFN-γ (5 ng/ml each) in human MSC spheroids cultured under dynamic conditions (small-scale). Compared to untreated spheroids, spheroids licensed with both IFN-γ and TNF-α exhibited increased IDO activity, increased IL6 secretion, and increased suppression of macrophage TNF-α secretion in a coculture assay. The effects were observed in spheroids cultured in FBS but not in a serum-free chemically defined medium (Zimmermann and McDevitt 2014). Although more studies on the biochemical preconditioning of MSC spheroids cultured under dynamic conditions are needed, the changes in the secretion of biomolecules with therapeutic potential observed after priming of static cultures may be promising for further advances in the production of media with improved potency for immunomodulation or regeneration.

### 6 Therapeutic Potential of Conditioned Medium Generated by 3D-Mesenchymal Stromal/Stem Cells in a Dynamic System

Enriched Mesenchymal Stromal/Stem Cells-derived conditioned medium as a new therapeutic strategy. Several *in vitro* studies and animal models of different pathologies have demonstrated the properties and regenerative potential of MSC-derived CM. It has been demonstrated that CM promotes tissue repair and exerts mitogenic, angiogenic, anti-inflammatory, immunomodulatory, antiapoptotic, and antitumor effects (Ferreira, Teixeira et al., 2018). In addition to these therapeutic effects, using a cell-free product such as CM has many advantages over direct MSCs administration. Considered a “cell-free therapeutic” (Sagaradze, Grigorieva et al., 2019), MSCs-derived CM does not require immune compatibility for the recipient to avoid rejection or strictly controlled sterile conditions for its administration, in contrast to cell-based treatments. In comparison to cell-based therapies, CM production requires less time for the expansion of millions of cells and has fewer costs for mass production (Gunawardena, Rahman et al., 2019). It also reduces the potential for tumour formation and embolism development associated with MSCs injection. CM can be manufactured and packaged as a frozen or lyophilized product and simplifies technical aspects associated with storage, transport, preservation, and availability. From a regulatory perspective, the biosafety, dose, and potency of CM can be evaluated in the same way as conventional biopharmaceutical agents, which facilitates its potential insertion into the market. Therefore, cell-free applications based on the components secreted by MSCs represent a rapidly developing and promising approach in regenerative medicine. However, it is necessary to establish a CM production protocol, including diverse operational aspects, to ensure that the content and the therapeutic outcomes of such CM products are homogeneous and consistent.

CM is a challenging sample to analyse, mainly because secreted proteins are quite diluted and often present at concentrations in the range of μg to ng. It has been reported that MSC-derived CM from standard 2D culture contains 217 ± 97 pg/ml VEGF, which is not sufficient, considering that a concentration of at least 5,000 pg/ml is required to induce angiogenesis *in vivo* (Bhang, Lee et al., 2014). This value reflects the low concentration of bioactive factors obtained from traditional culture conditions. Moreover, a systematic review of various studies on MSC-derived CM applied in models of various diseases determined that MSCs from the same source yielded different concentrations of factors (Pawitan 2014). Although these differences occur because of the different cell donors involved (Assoni, Coatti et al., 2017), several culture parameters can be optimized to develop a novel large-scale productive strategy. Such a production strategy, as noted above, can be achieved under 3D dynamic culture conditions.

### 6.1 Increased Levels of Secreted Factors in Conditioned Medium Derived From Dynamic 3D Mesenchymal Stromal/Stem Cells Cultures

In the literature, few works characterize the CM and do so in an unexhaustive manner. According to our review, Table 2 summarizes the work done in this area. It is observed that both the quantified paracrine factors and the identification of EVs are limited to the particular aim of the research and there are no studies in which a large-scale exploratory analysis of its components is carried out. In addition, the secretion profile is mainly based on the presence of three–five secreted factors, considering more frequently those with a proangiogenic potential (VEGF, FGF2, HGF and TGFβ1). Analysis of CM3D obtained from dynamic cultures showed that compared to CM derived from 2D culture (CM2D), CM3D potentiates the secretion of proangiogenic factors such as VEGF, FGF2, HGF, CXCL2 and CXCL12 to a great extent (Bhang, Lee et al., 2014; Kwon, Bhang et al., 2015). Some studies reported enormous increases, such as a 15-fold increase in FGF2 and an 80-fold increase in VEGF (Santos, Camoes et al., 2015). Moreover, the secretion of these factors is enhanced by 1% hypoxia (Bhang, Cho et al., 2011). It has also been observed that 3D culture induces the secretion of immunomodulatory factors. Significant increases of up to 6-fold for TGFβ (Cho, Song et al., 2012; Zimmermann and McDevitt 2014; Santos, Camoes et al., 2015) and up to 4-fold for PGE2 and IL6 have been detected (Zimmermann and McDevitt 2014).

### 6.2 CM3D and *In Vivo* Models

The therapeutic potential of CM3D has been studied in some *in vivo* models. Most of them are based on tissue repair promoted by angiogenesis. Regardless of whether the spheroids were performed in hanging drops or formed directly in agitation systems, their CM possessed high angiogenic potential when compared to that of CM2D. It has been shown that CM3D promotes neovascularization and structural and functional recovery in models of cerebral stroke (Cho, Song et al., 2012) and hindlimb ischaemia (Bhang, Lee et al., 2014). Additionally, the higher concentration of angiogenic factors in CM3D potentiated wound healing and skin regeneration (Kwon, Bhang et al., 2015; Santos, Camoes et al., 2015). In addition to models of tissue damage, CM3D has been administered in models of proinflammatory diseases, such as arthritis. CM3D exerted a positive effect on chronic arthritis by inducing chondrocyte migration and ameliorating synovial inflammation and bone erosion (Miranda, Camoes et al., 2019). This recovery was due to the increased secretion of the trophic factors PDGF-BB, FGF2, CCL1, SCF-1, and GMCSF, which participate in tissue repair, and the anti-inflammatory cytokines IL10 and LIF. In addition to this arthritis model, it would be interesting to test the effects of CM3D in models of autoimmune or proinflammatory diseases and to analyse the secretion of immunosuppressive factors such as PGE2 and TSG6.

### 6.3 Extracellular Vesicles and Dynamic 3D Culture

EVs and their bioactive cargoes are an emerging issue in the context of cell-free therapies. EVs are membrane-surrounded structures that are released by all cells and are mediators of intercellular communication, serving as vehicles for the transfer and exchange of enzymes, cytokines, chemokines, immunomodulatory and growth factors, lipids, mRNA, and miRNA. EVs are classified according to their size and origin. Among these types of EVs, the most abundant are exosomes and microvesicles. Exosomes are the smallest EVs, with an approximate diameter of 30–100 nm, and they originate in the endosome, while microvesicles have a diameter of 50–1,000 nm and are shed directly from the cell membrane (Raposo and Stoorvogel 2013).

Similar to other cell types, MSCs also release EVs. The first observations on the protective effect of MSC-derived EVs (MSCs-EVs) were obtained in models of acute renal failure (Bruno, Grange et al., 2009) and myocardial ischaemia (Lai, Arslan et al., 2010). Many experimental animal models have shown that MSC-EVs exert a potent regenerative effect in stroke, traumatic brain injury, pulmonary hypertension, and wound healing (Börger, Bremer et al., 2017). Both local and systemic administration of MSCs-EVs modulates and attenuates autoimmune and inflammatory diseases, as has been proven in colon inflammation, liver damage and fibrosis, neuroinflammatory disease, and inflammatory eye disease (Harrell, Fellabaum et al., 2019). The worldwide increase in the number of severe COVID-19 patients has prompted the development of phase II clinical trials to treat these patients with exosomes. The ExoFlo biopharmaceutical product, which is composed of exosomes and growth factors, decreased lung inflammation and was safe, without adverse effects (Sengupta, Sengupta et al., 2020). The therapeutic effects elicited by MSC-EVs are as potent as or even more potent than those observed after transplantation of MSCs.

The low yield of EVs from CM has led efforts to develop strategies to increase the production of MSC-EVs. One alternative is the manipulation of key genes involved in exosome biogenesis. However, manipulation of cell culture conditions to enhance EV production has the additional advantage of being scalable. Moreover, the biogenesis and release of MSCs-EVs are regulated according to the conditions of the cellular microenvironment conditions, which can be used to maximize their therapeutic potential. Preconditioning approaches can induce protein, RNA, and cytokine profiles different from those of EVs from naïve MSCs. Early studies showed that exosomes derived from MSCs primed by hypoxia increased migration and promoted tube formation in endothelial cells (Salomon, Ryan et al., 2013). Further studies have shown that they also promote tissue regeneration and exert neuroprotective effects (Joo, Suh et al., 2020). Preconditioning of MSCs with proinflammatory cytokines yields exosomes enriched in anti-inflammatory mediators, anti-inflammatory miRNAs, and neuroprotective proteins. These exosomes promoted the transformation of regulatory T cells *in vitro* and cartilage tissue repair in an OA model (Domenis, Cifù et al., 2018; Harting, Srivastava et al., 2018; Zhang, Fu et al., 2018).

Increasing the production and release of EVs from MSCs is an urgent clinical need. However, studies on large-scale production are still limited. According to a worldwide survey on EV isolation techniques, only 77% of respondents used less than 100 ml of CM (Gardiner, Di Vizio et al., 2016). Generating this volume of starting material via standard 2D culture techniques is insufficient for a market-scale product. Therefore, preconditioning approaches that increase EV generation are critical. Consistent with this need, dynamic cultures in bioreactors that allow the generation of large volumes of CM enriched in EVs are a promising platform. Recently, spheroids preformed in microwells and cultured for 7 days with orbital agitation showed an increase of over 100-fold in the number of MSC-EVs secreted into the CM compared to that observed after 2D culture (Cha, Shin et al., 2018). These EVs obtained from 3D cultures exhibited increased levels of cytokines and miRNAs related to immunomodulation, angiogenesis, and neurogenesis. Continuing with this strategy, it would be interesting to study the composition of EVs under different dynamic culture conditions and with modulation of different operational variables, considering different agitation speeds, the composition of the culture medium, pH, and temperature. When manufacturing challenges have been resolved, it will be possible to assure greater consistency of the final product to advance these therapies to the bedside.

## Conclusion and Perspectives

Here, we have discussed the culture of MSCs in 3D dynamic support-free systems to obtain CM enriched in paracrine factors and microvesicles with therapeutic potential, either by inducing tissue repair or decreasing inflammation. Although the 3D dynamic culture has not yet replaced 2D methods on a large scale, current data show that CM obtained from these systems is a promising cell-free alternative for future therapeutic applications. Nevertheless, no clinical trials have evaluated the efficacy of MSC spheroids and derived CM in clinical settings. For this reason, it is necessary to develop processes to obtain CM as a safe and effective product derived from a highly reproducible and routinely applicable bioprocess based on dynamic bioreactors. To do this, it would be interesting to characterize and establish the secretion profiles of the CMs, using a minimum set of paracrine factors and secreted microvesicles, which allow the prediction of the potential therapeutic effect of the CM.

On the other hand, as evidenced in this work, the dynamic cultivation of MSCs in bioreactor systems provides various possibilities to establish operational conditions that could have a substantial impact on obtaining cell-free products enriched by secreted factors. However, to date, many of these variables have not been studied in depth for CM production. The consideration of this type of environmental variables in subsequent work could be a great contribution to this area of research. As the next step in obtaining large-scale batch CM as a clinical product, applying conventional protein identification and proteomics techniques to a sample as complex as CM often results in high losses and low recovery. Thus, it is essential to address the production of CM with a multidisciplinary approach that brings together current knowledge and techniques regarding the properties of MSCs, bioengineering, bioprocesses, and pharmaceutical technology.

## Abbreviations

AD, adipose tissue; BM, bone marrow; CM, conditioned medium; CM3D, CM derived from dynamic 3D MSC cultures; CM2D, CM derived from dynamic 2D MSC cultures; CEM, Iscove’s modified Dulbecco’s medium; CRM, clinically relevant medium composed of amino acids, vitamins and glucose; EVs, MSC-derived EVs; FBS, fetal bovine Serum; HS, Horse serum; ia, Is assumed; MVs, microvesicles; MSCs, Mesenchymal stromal/stem cells; nr, Not reported; OT, oxygen tension; Ps, cell passage; RWV, rotating wall vessel; rpm, revolutions per minute; SF, stirring flasks; STR, stirred tank reactor; UC, umbilical cord; SyF, synovial fluid; (1), 1 month; (2), 2 months; 3D, Three-dimensional cultures; 2D, two-dimensional cultures.
